# A Non-canonical Wnt Signature Correlates With Lower Survival in Gastric Cancer

**DOI:** 10.3389/fcell.2021.633675

**Published:** 2021-04-01

**Authors:** Pablo Astudillo

**Affiliations:** Facultad de Ciencias de la Salud, Instituto de Ciencias Biomédicas, Universidad Autónoma de Chile, Santiago, Chile

**Keywords:** Wnt, gastric cancer, Wnt5a, Frizzled-7, Frizzled-2, Ror2, EMT

## Abstract

Genetic evidence suggests a role for the Wnt/β-catenin pathway in gastric cancer. However, Wnt5a, regarded as a prototypical non-canonical Wnt ligand, has also been extensively associated with this disease. Therefore, the roles of the Wnt signaling pathway in gastric cancer initiation and progression, and particularly the precise mechanisms by which the non-canonical Wnt pathway might promote the development and progression of gastric cancer, are not entirely well understood. This article analyzes publicly available gene and protein expression data and reveals the existence of a *WNT5A*/*FZD2*/*FZD7*/*ROR2* signature, which correlates with tumor-infiltrating and mesenchymal cell marker expression. High expression of *FZD7* and *ROR2* correlates with a shared gene and protein expression profile, which in turn correlates with poor prognosis. In summary, the findings presented in this article provide an updated view of the relative contributions of the Wnt/β-catenin and non-canonical Wnt pathways in gastric cancer.

## Introduction

The Wnt signaling pathway plays roles during embryonic development and adult homeostasis, and perturbation of this pathway is linked to several diseases (Logan and Nusse, 2004). This pathway is commonly divided into two main branches. One depends on the stabilization of β-catenin, which acts as a transcriptional co-regulator (Valenta et al., 2012). Thus, this pathway is commonly known as “Wnt/β-catenin” (or “canonical”) pathway. On the other hand, there is a subset of Wnt pathways that are independent of β-catenin, referred to as “non-canonical” (or “Wnt/β-catenin independent”) pathways (Semenov et al., 2007), which activate intracellular effectors, modulating cell behavior (Schlessinger et al., 2009). Aberrations in both pathways have been linked to cancer initiation and progression (Anastas and Moon, 2012; Kikuchi et al., 2012).

Gastric cancer (GC) accounts for approximately 8% of total cancer-related deaths (Bray et al., 2018). Therefore, understanding which pathways are involved in the initiation and progression of GC is of paramount relevance. In this regard, mutations in genes encoding components of the Wnt/β-catenin pathway, such as *CTNNB1*, *APC*, or *RNF43*, have been reported in genetic studies (Cancer Genome Atlas Research Network, 2014; Cristescu et al., 2015). However, Wnt5a, a ligand that usually activates non-canonical Wnt signaling, is also overexpressed in GC patients (Saitoh et al., 2002; Boussioutas et al., 2003; Kurayoshi et al., 2006; Li et al., 2014; Nam et al., 2014). Therefore, the role of the Wnt signaling pathways in GC initiation and progression remains to be fully clarified.

Canonical Wnt ligands act by binding to frizzled class receptors and the LDL receptor related protein 5 and 6 (LRP5/6) co-receptors, while non-canonical ligands, such as Wnt family member 5A (Wnt5a), signal by binding to frizzled receptors and co-receptors such as receptor tyrosine kinase-like orphan receptor 1 and 2 (ROR1/2) and receptor-like tyrosine kinase (RYK), among others (Niehrs, 2012). Mechanistically, Wnt5a has been linked to integrin-adhesion turnover and thus to enhanced migration and invasion (Matsumoto et al., 2010). In GC, Wnt5a has also been associated with cell migration and invasion (Kurayoshi et al., 2006; Yamamoto et al., 2009; Hanaki et al., 2012; Shojima et al., 2015). Consequently, blocking Wnt5a signaling leads to impaired GC cell tumorigenesis (Yamamoto et al., 2009; Hanaki et al., 2012; Shojima et al., 2015). However, most reports studying Wnt5a in GC were carried out before the release, or recent update, of cancer gene and protein expression and analysis databases, such as cBioPortal (Cerami et al., 2012; Gao et al., 2013), TIMER (Li T. et al., 2017), GEPIA (Tang et al., 2017, 2019), and The Cancer Proteome Atlas (TCPA) (Li et al., 2013; Li J. et al., 2017). Therefore, an updated view regarding the relevance of the non-canonical Wnt pathway in GC might provide additional or improved evidence for its role in this disease, lending support to future studies exploring the molecular mechanisms linking this pathway to GC progression and possible pharmacological approaches.

Consequently, the main goal of this report is to analyze the association between non-canonical Wnt components and GC prognosis using publicly available data. This article studies the possible association of *WNT5A*, *FZD2* (encoding the frizzled class receptor 2), *FZD7* (encoding the frizzled class receptor 7), and *ROR2* (encoding the co-receptor ROR2) with GC, revealing a potential gene signature correlating with poor prognosis. This article also compares the relationship between canonical and non-canonical Wnt signatures with GC prognosis, aiding to clarify the relative contribution of each Wnt signaling branch to the progression of this disease.

## Materials and Methods

### Cancer Gene and Protein Expression Data Analysis

Gene expression was studied using GEPIA (version 2.0) and cBioPortal. In GEPIA, the box-plot function was used to compare tumor (T) versus normal (N) expression, matching TCGA stomach adenocarcinoma (STAD) tumor with normal and GTEx data. The expression data were log_2_ (TPM + 1) transformed, and the log_2_FC was defined as the median (T)—median (N). Differential analysis was performed using one-way ANOVA. In cBioPortal, Onco Query Language was used to query for samples with specific expression values, as indicated in the main text. For mRNA (RNA Seq V2 RSEM) and protein expression, z-score thresholds = ±1 were used. The STAD Firehose Legacy dataset was used in all analyses. All data was collected between July and November 2020.

To estimate the correlation between genes and the infiltration of immune cells or epithelial-to-mesenchymal transition (EMT) markers, two approaches were followed. First, the correlation between gene signatures was assessed in GEPIA, using the “Correlation Analysis” tool, comparing STAD Tumor data with STAD Normal plus stomach GTEx data, and computing the Spearman correlation coefficient. Second, the “Gene” module from TIMER (version 1.0) was employed to assess immune cell infiltration. Immune (Wei et al., 2020) and epithelial/mesenchymal (Xue et al., 2020) gene signatures were used to assess the correlation between non-canonical gene signatures and immune infiltration or EMT, respectively. The markers employed are listed in Supplementary Table 1.

To search for proteins whose expression correlated with *FZD7* or *ROR2* in cBioPortal, a z-score threshold of ± 1.0 was used for both mRNA (RNA Seq V2 RSEM) and protein expression, using samples with protein expression data (392 samples). Functional interaction data for Wnt5a was queried in STRING (Szklarczyk et al., 2019), using only “Experiments” and “Databases” interaction sources. A network showing the twenty-first interactors was generated, with the edges depicting the strength of data support and with a minimum interaction score of 0.4.

### Survival Analysis

For gene expression, Kaplan-Meier plots were obtained using GEPIA and KM Plotter (Szász et al., 2016). In GEPIA, the *WNT5A*/*FZD2*/*ROR2* and *WNT5A*/*FZD7*/*ROR2* signatures were assessed for overall survival (OS) and disease-free survival (RFS) by using the “Gene Signature” option, maintaining the remaining parameters as default, using either the upper (66.7%) and lower (33.3%) terciles, or the upper (75%) and lower (25%) quartiles, as indicated. Alternatively, survival plots were generated using the “gastric cancer” option in KM Plotter, using terciles and the best probe for each gene (*WNT5A*, 213425_at; *ROR2*, 205578_at; *FZD2*, 238129_s_at; *FZD7*, 203706_s_at). The mean expression of the selected genes was used for the analysis, computing the Spearman correlation and excluding the GSE62254 dataset, with all other parameters set as default.

To assess the correlation between protein expression and survival, data from The Cancer Proteome Atlas (TCPA, version 3.2) was retrieved using the STAD dataset for analysis (392 samples). The univariant Cox and Log-rank test p-values for each protein are shown together with each plot, with samples separated into “high” and “low” expression groups by the median value of protein expression.

### Functional Enrichment Analysis

To search for gene ontology (GO) terms over-represented among the genes correlating with *FZD7* and *ROR2* expression, the list of genes with correlation values > 0.5 was downloaded from cBioPortal. A curated list (see main text) was then analyzed, employing the WEB-based GEne SeT AnaLysis Toolkit (WebGestalt) website (Liao et al., 2019), using the “Over-Representation Analysis” option, selecting “Genome” as the reference set, and using the “Biological Process noRedundant,” “Cellular Component noRedundant,” and “Molecular Function noRedundant” terms. ENSEMBL gene IDs were used for the analysis, while the remaining parameters were set as default. Gene names were converted to ENSEMBL IDs with SYNGO (Koopmans et al., 2019). The same gene list was used for an enrichment analysis in Metascape (Zhou et al., 2019). The GO “Biological Process,” “Cellular Component,” and “Molecular Function,” plus the “GOSlim,” “Protein Function,” and “Canonical Pathways” categories were employed, using the custom analysis option and maintaining the default parameters. The Venn diagram was created using an online tool^1^.

## Results

### Expression of Wnt Components in GC

**FIGURE 1:**
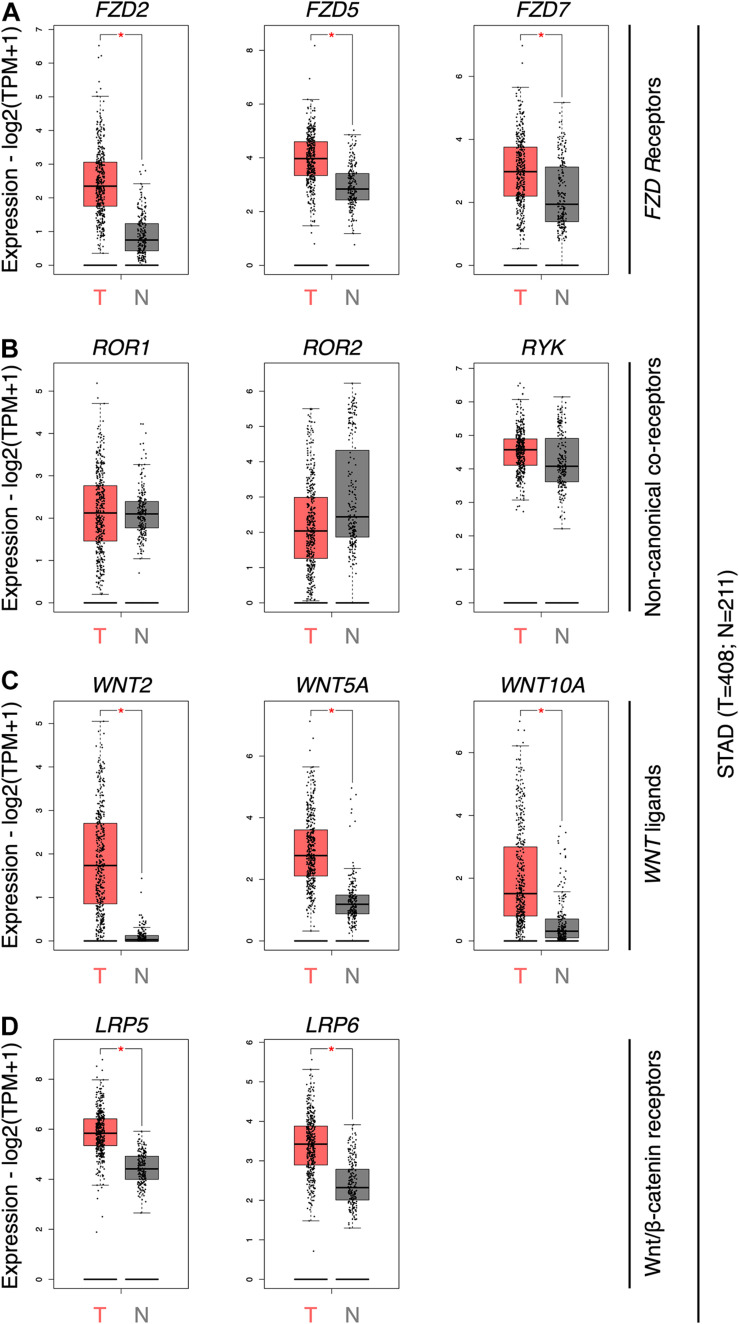
Gene expression profile for selected WNT components in GC. **(A)** The abundance of *FZD2*, *FZD5*, and *FZD7* genes in GC tumor tissue (T; *n* = 408) and normal adjacent tissue and matched GTEx data (*N*; *n* = 211) samples. **(B)** Abundance of *ROR1*, *ROR2*, and *RYK* genes; **(C)** *WNT2*, *WNT5A*, and *WNT10A* genes; and **(D)** *LRP5* and *LRP6* genes. Differential expression is calculated by one-way ANOVA. Expression data is shown as log2(TPM + 1); TPM, transcripts per million. *, differentially expressed genes.

To obtain an updated view of the role of the Wnt pathway in GC, the expression of the ten Frizzled receptors (*FZD* genes), several Wnt co-receptors (*ROR1*, *ROR2*, *RYK*, *LRP5*, and *LRP6*), and *WNT* genes were first assessed using GEPIA. *FZD2*, *FZD5*, and *FZD7* was significantly overexpressed in GC samples (Figure 1A). The expression of all 19 *WNT* genes was then evaluated (Figure 1B). *WNT5A*, *WNT2*, and *WNT10A* were the only ligands overexpressed in GC samples. Wnt family member 2 (Wnt2) is expressed in GC, correlating with nuclear β-catenin, loss of E-cadherin, and enhanced migration and invasion (Katoh, 2001; Cheng et al., 2005; Zhang et al., 2018). Wnt family member 10A (Wnt10a), on the other hand, remains poorly characterized in GC (Kirikoshi et al., 2001a). The non-canonical co-receptors *ROR1*, *ROR2*, and *RYK* did not show differences between both conditions (Figure 1C). In contrast, the canonical co-receptors *LRP5* and *LRP6* were overexpressed in GC (Figure 1D).

As previously noted, Wnt5a has been extensively related to GC. Wnt5a functionally interacts with several frizzled receptors, including frizzled class receptor 2 (FZD2) and frizzled class receptor 7 (FZD7) (Supplementary Figure 1). Wnt5a signals through FZD2 (Hanaki et al., 2012). On the other hand, FZD7 is expressed in the gastric epithelium (Flanagan et al., 2017) and is overexpressed in GC (Kirikoshi et al., 2001b). FZD7 has been shown to play a role in GC (Flanagan et al., 2019), and it might be involved in Wnt/β-catenin signaling in this disease (Li et al., 2018). Consequently, the expression of *WNT5A*, *FZD2*, *FZD7*, and the non-canonical co-receptors *ROR1*, *ROR2*, and *RYK* was further explored using cBioPortal. These non-canonical co-receptors were included since they might be required for optimal Wnt signaling, even if not being overexpressed. For comparison, the canonical co-receptors *LRP5* and *LRP6*, as well as *WNT2*, were also included.

Automated clustering of samples using cBioPortal shows overlap among cases with high levels of several genes, although some groups do appear (see Supplementary Figure 2 for an extended analysis). It is worth noting that cases with high *WNT5A* and *WNT2* expression partially overlap. Regarding *WNT5A*, its precise source in GC has not been defined (Astudillo, 2020). For instance, some GC cell lines show low *WNT5A* levels, while cancer-associated fibroblasts (CAFs) or tumor-associated macrophages (TAMs) might secrete Wnt5a in the context of GC (Zhao et al., 2013; Wang et al., 2016). Therefore, observations regarding Wnt5a must be interpreted with caution. Nonetheless, since Wnt5a inhibits Wnt/β-catenin signaling in different contexts (Torres et al., 1996; Ishitani et al., 2003; Topol et al., 2003; Westfall et al., 2003; Mikels and Nusse, 2006), GC cases with high *WNT5A* and *WNT2* levels are likely characterized by non-canonical Wnt signaling, although this hypothesis requires corroboration.

### A Non-canonical Wnt Gene Signature Correlates With Lower Survival in GC

**FIGURE 2:**
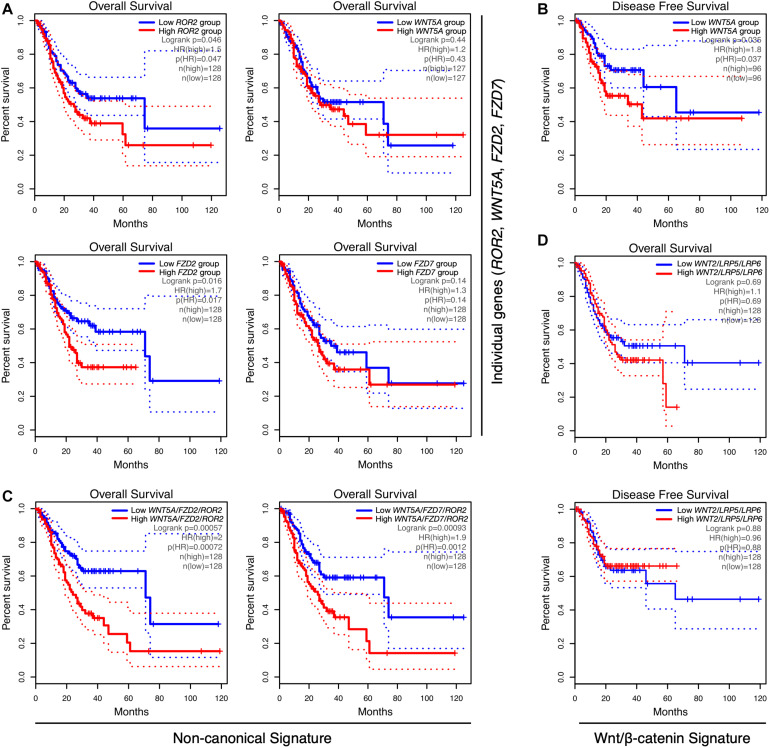
Correlation between gene expression and overall survival in GC. **(A)** Correlation between *ROR2*, *WNT5A*, *FZD2*, and *FZD7* gene expression and overall survival (OS), using terciles, in GEPIA. **(B)** Correlation between low and high *WNT5A* expression and disease-free survival (RFS), using quartiles. **(C)** Correlation between the *WNT5A*/*FZD2*/*ROR2* (left) and *WNT5A*/*FZD7*/*ROR2* (right) gene signatures and OS, using GEPIA. **(D)** Correlation between the Wnt/β-catenin gene signature *WNT2*/*LRP5*/*LRP6*, and OS (top) or RFS (bottom), by using terciles. The number of samples per condition, denoted as n(high) (red lines) and n(low) (blue lines), as well as the HR and Log-rank *p*-values, are indicated in each plot. Dotted lines show the 95% confidence interval (CI).

Next, the correlation between Wnt-related genes and clinical outcome was assessed. Notably, although *ROR2* levels were not increased in tumor samples (Figure 1), high *ROR2* expression was correlated with lower OS (Figure 2A; HR = 1.5; Log-rank *p* = 0.046). A correlation between high expression and lower OS was also observed for *FZD2* (Figure 2A; HR = 1.7; Log-rank *p* = 0.016). No significant correlation was observed for *FZD7* and *WNT5A* (Figure 2A) and OS by using terciles. For *WNT5A*, however, a significant correlation between high levels and lower RFS was observed using quartiles (Figure 2B). Of note, previous studies have correlated *WNT5A* expression with survival, as well as with other clinical parameters (Nam et al., 2017). Therefore, Wnt5a likely plays a vital role in a subset of cases. On the contrary, and despite their high levels in GC samples, increased expression of *WNT2*, *LRP5*, or *LRP6* did not show a significant correlation with lower OS (see Supplementary Table 2 for OS using terciles) or RFS, by using terciles or quartiles (data not shown).

The current model for Wnt signaling emphasizes the relevance of the cellular context in determining the signaling outcome. Following this notion, it is possible to speculate that most cases with high levels of LRP5/6 co-receptors likely represent a particular GC subset, which might be sensitive to increased levels of canonical Wnt ligands, particularly Wnt2. In contrast, cases with high levels of frizzled receptors might be sensitive to increased Wnt5a levels, for instance, secreted by CAFs or TAMs, even in the presence of normal levels of non-canonical co-receptors such as ROR2. Thus, it was hypothesized that increased levels of Wnt5a plus either FZD2 or FZD7, in the context of normal ROR2 levels, should activate non-canonical Wnt signaling. If this is the case, then a combined *WNT5A*/*FZD2*/*ROR2* or a *WNT5A*/*FZD7*/*ROR2* gene signature might be correlated with a worsened prognosis.

Consequently, the correlation between these non-canonical Wnt signatures and clinical outcome was analyzed. As expected, there was a dramatic decrease in OS in the high expression terciles for both signatures (Figure 2C). High *WNT5A*/*FZD2*/*ROR2* expression was also correlated with lower RFS using terciles (*n* = 128 per group; HR = 1.9; Log-rank *p* = 0.0088; Supplementary Figure 3A). High expression of the *WNT5A*/*FZD7*/*ROR2* signature was not correlated with lower RFS using terciles; however, it was significantly correlated when quartiles were chosen (*n* = 96 per group; HR = 1.9; Log-rank *p* = 0.027; Supplementary Figure 3B). Notably, a decrease in the HR is observed when *WNT5A* is excluded from both gene signatures (Supplementary Table 2). Therefore, and considering the high expression of *WNT5A* in GC samples, this gene was included in the signatures for subsequent analyses. An independent assessment using KM Plotter also revealed a lower OS in the high expression group for both signatures (Supplementary Figures 3C,D).

Importantly, the *WNT2*/*LRP5*/*LRP6* canonical Wnt signature did not significantly correlate with either OS or RFS, using terciles (Figure 2D and Supplementary Table 2, lines 10 and 17) or quartiles (Supplementary Table 2, lines 18 and 19). Including *FZD2* or *FZD7* in the *WNT2*/*LRP5*/*LRP6* signature modestly increased the correlation with lower OS. Still, this correlation is lower than those observed for the non-canonical Wnt signatures (compare lines 20 and 21 with lines 1 and 4 in Supplementary Table 2). Collectively, this data suggests that *WNT5A*, the receptors *FZD2*, *FZD7*, and the co-receptor *ROR2* might define two gene signatures correlated with poor prognosis.

### Non-canonical Wnt Signatures, Tumor-Infiltrating Immune Cells, and EMT

The presence of tumor-infiltrating immune cells (TIICs) in the tumor microenvironment is usually associated with poor prognosis (Fridman et al., 2012) and has been characterized recently in GC (Li et al., 2019; Wang et al., 2020; Wei et al., 2020; Wu et al., 2020). To determine if the non-canonical Wnt signatures are correlated with immune cell infiltration, the correlation between the two gene signatures and markers of specific immune cell types was assessed using GEPIA (“signature-to-signature” comparison). Previous reports have shown higher infiltration of macrophages in GC (Wei et al., 2020); therefore, the analysis below focuses on the correlation between the gene signatures and markers of monocytes, tumor-associated macrophages (TAMs), and M1/M2 macrophages (listed in Supplementary Table 1).

**FIGURE 3:**
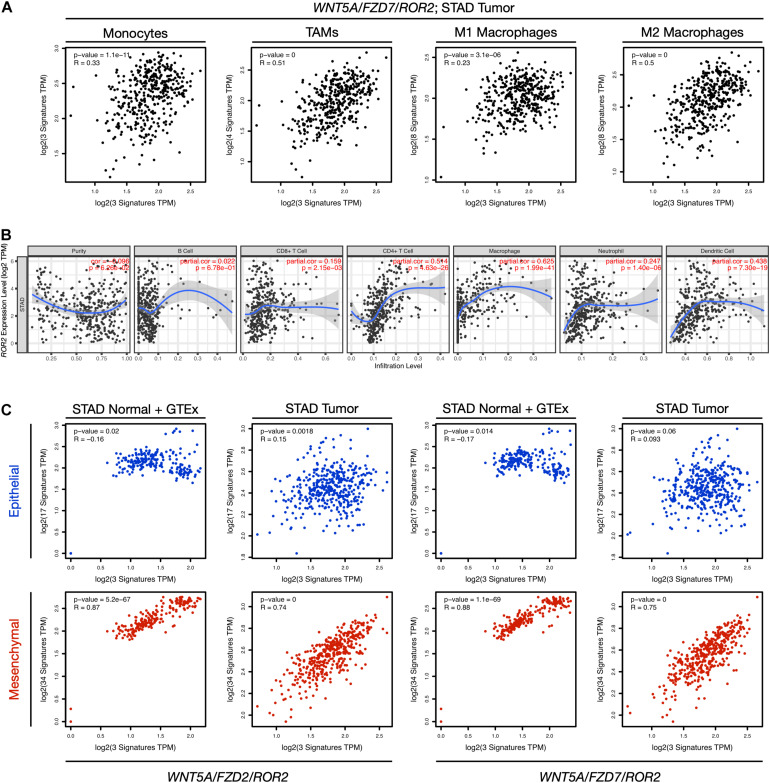
Correlation between gene expression, immune cell markers, and EMT. **(A)** The correlation between the *WNT5A*/*FZD7*/*ROR2* signature and signatures of monocytes, tumor-associated macrophages (TAMs), M1 macrophages, and M2 macrophages for the STAD Tumor dataset was evaluated using the “Gene Signature” comparison option in GEPIA (Correlation Analysis). The Spearman correlation coefficients and *p*-values are indicated in each plot. The markers used for the analysis are listed in Supplementary Table 1, and a full comparison including all Wnt signatures and both “STAD Normal + GTEx data” and “STAD Tumor data” is presented in Supplementary Table 3. **(B)** Correlation between *ROR2* gene expression and immune cell infiltration. The analysis was performed using TIMER, adjusting by tumor purity. See Supplementary Figure 4 for a panel including *WNT5A*, *FZD2*, and *FZD7*. **(C)** Correlation between the indicated non-canonical Wnt signatures and gene signatures corresponding to an epithelial (blue) or mesenchymal (red) transcriptional program. Both “STAD Normal + GTEx data” and “STAD Tumor data” are included in the analysis. See main text for details. TPM, transcripts per million.

When STAD normal and GTEx tissue data was employed, there was a significant correlation between the non-canonical Wnt gene signatures and all cell types (Supplementary Table 3), which was more evident for TAMs and M2 macrophages (see lines 2a, 4a, 6a, and 8a in Supplementary Table 3). Surprisingly, these correlations were decreased when using STAD tumor data (see Figure 3A for STAD tumor data and the *WNT5A*/*FZD7*/*ROR2* signature; see Supplementary Table 3 for full comparisons, and Supplementary Figure 4 for an extended discussion), and similar observations were made for both non-canonical Wnt signatures (Supplementary Table 3). Importantly, when the *WNT2*/*LRP5*/*LRP6* canonical Wnt signature was used for analysis using STAD tumor data, the modest correlation with TAMs (compare lines 2b, 6b, and 10b in Supplementary Table 3) and M2 markers (compare lines 4b, 8b, and 12b in Supplementary Table 3) was lost, favoring a role for the non-canonical Wnt pathway in macrophage infiltration.

Subsequently, each gene was assessed independently for their correlation with immune cell infiltration using TIMER as an alternative approach. *WNT5A* and *FZD2* did not correlate with immune infiltration, while *FZD7* showed a weak correlation with CD4+ T cell and macrophage infiltration (Supplementary Figure 4). However, *ROR2* showed a stronger correlation with CD4+ T cell and macrophage infiltration (Figure 3B).

Collectively, these results prompted the question of whether these immune signatures were correlated with GC prognosis. Interestingly, a modest correlation (HR = 1.5; Logrank *p* = 0.047) was observed only for an M2 gene signature and OS (Supplementary Table 4).

The analysis focused next on EMT, a process that correlates with cancer initiation and progression (Dongre and Weinberg, 2019), including in GC (Peng et al., 2014). Recently, a comprehensive analysis of developmental signaling pathways (including the non-canonical Wnt pathway), and their correlation with EMT was reported (Xue et al., 2020). The epithelial and mesenchymal signatures reported by this study (Supplementary Table 1) were used to corroborate whether the non-canonical Wnt signatures correlated with either an epithelial or mesenchymal transcriptional program. For both signatures, there was a strong correlation with the mesenchymal, but not the epithelial, transcriptional program (Figure 3C). Of note, the correlation between the *WNT2*/*LRP5*/*LRP6* signature and the mesenchymal signature was decreased when using STAD tumor data, compared to the non-canonical signatures (Supplementary Figure 5A). Importantly, only the mesenchymal signature was correlated with lower OS (HR = 1.9; Log-rank *p* = 0.0015; Supplementary Figure 5B). Altogether, this analysis suggests that Wnt5a signaling, mediated by FZD2 and FZD7, together with the co-receptor ROR2, might be correlated with EMT and, to a lesser extent, with macrophage infiltration, which might partly explain their correlation with poor prognosis.

### Analysis of the Expression Profile Associated With FZD7 and ROR2

**FIGURE 4:**
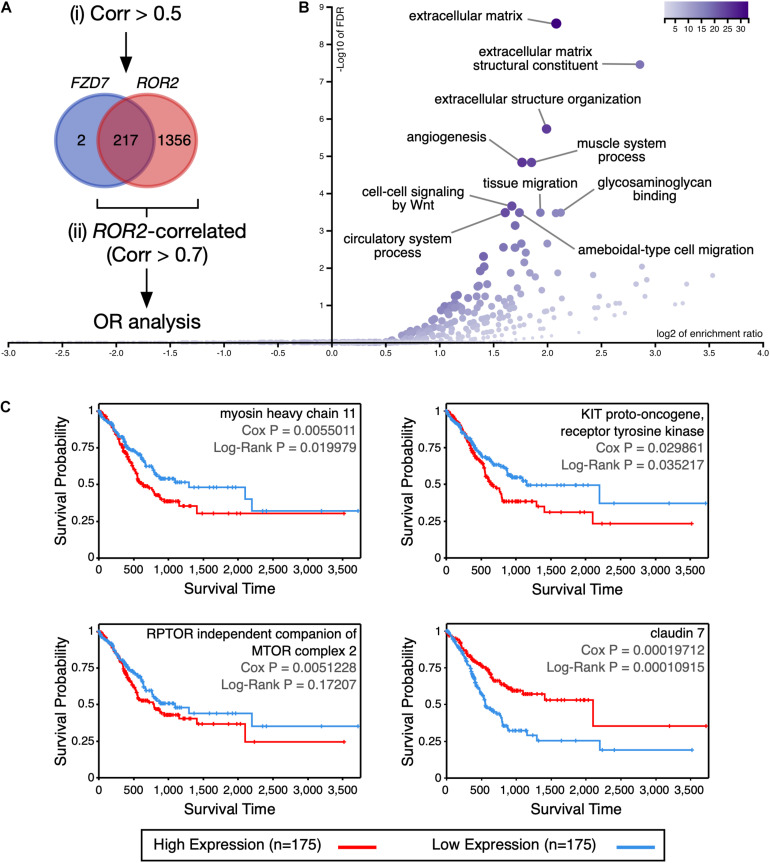
Analysis of genes and proteins correlated with *FZD7*/*ROR2* expression. **(A)** Venn diagram showing the overlap between the genes positively correlated with either *FZD7* or *ROR2* gene expression. For simplicity, the size of the circles is not proportional to the number of genes. The rationale of the analysis is summarized in the diagram. **(B)** Volcano plot showing significantly over-represented (OR) Gene Ontology categories, according to WebGestalt. **(C)** Correlation between myosin heavy chain 11, KIT proto-oncogene, receptor tyrosine kinase, RPTOR independent companion of MTOR complex 2, and claudin 7 protein expression and survival, according to the TCPA database.

To determine the molecular processes that might be differentially regulated in GC patients with high expression of *FZD7* and *ROR2*, differences in expression at both the gene and protein level were analyzed using cBioPortal. The analysis focused on *FZD7* because this receptor might have a predominant role in GC (see Discussion). Genes with a Spearman’s correlation value > 0.5 relative to either *FZD7* or *ROR2* expression were first retrieved since genes correlating with *FZD7* expression had low correlation values (219 genes; Supplementary Table 5). Of note, 217 out of the 219 genes correlated with *FZD7* expression were contained in the *ROR2*-correlated list (1573 genes; Figure 4A and Supplementary Table 5). To obtain a more stringent view of the processes correlated with high *FZD7*/*ROR2* signaling, genes correlating with *ROR2* using a cut-off value > 0.7 were further analyzed. This list, containing 278 genes (Supplementary Table 5), was used for an over-representation analysis in WebGestalt and Metascape. GO terms related to extracellular matrix constitution and organization, as well as terms related to cell migration, are among the top over-represented terms, according to WebGestalt (Figure 4B). Analysis using Metascape revealed additional over-represented terms (Supplementary Figure 6). The two methods also identified GO terms related to cell signaling.

Next, proteins correlating with either *FZD7* or *ROR2* expression and with a Spearman correlation value < −0.3 and > 0.3 were retrieved and analyzed. It must be noted that the TCGA contains RPPA (Reverse-Phase Protein Array) data only for nearly 300 proteins (Li J. et al., 2017). Six proteins were positively correlated with *FZD7* expression, while 29 proteins were positively correlated with *ROR2* expression (Supplementary Table 6). Mimicking the trend observed at the mRNA level, the six proteins correlated with *FZD7* expression were contained in the list of proteins correlated with *ROR2* (Supplementary Table 6). Among these, only activin A receptor like type 1 (ACVRL1), KIT proto-oncogene, receptor tyrosine kinase (KIT), myosin heavy chain 11 (MYH11), and RPTOR independent companion of MTOR complex 2 (RICTOR) showed a *p*-value < 0.05 in the *ROR2*-correlated list. Importantly, high protein expression of KIT and MYH11 correlated moderately with lower survival, while a similar trend was observed for RICTOR (Figure 4C). For KIT and MYH11, a moderate correlation with the mesenchymal signature was observed (Supplementary Figures 7A,B).

For proteins negatively correlated with *FZD7* or *ROR2* mRNA expression, eight and 16 proteins were found, respectively. Again, seven out of the eight proteins negatively correlated with *FZD7* were contained in the *ROR2* list (Supplementary Table 6). None of these proteins showed a *p*-value < 0.05 in the *ROR2*-correlated list. However, low protein levels of claudin 7 (CLDN7) correlated with lower survival (Figure 4C). In agreement, low *CLDN7* expression correlates with lower OS using quartiles (Supplementary Figure 8). In summary, *FZD7* and *ROR2* expression correlate with an expression profile with potential prognosis value in GC.

### Discussion and Conclusion

Here, publicly available gene expression and clinical data were analyzed to provide an updated view about the relative contribution of the canonical and non-canonical branches of the Wnt pathway to GC prognosis. Mutations in Wnt/β-catenin pathway genes are usually associated with tumor initiation (Anastas and Moon, 2012). On the other hand, the non-canonical Wnt pathway is involved in GC cell migration and invasion (Kikuchi et al., 2012). However, most reports addressing potential mechanisms by which the non-canonical Wnt pathway might be involved in GC are limited to animal models and *in vitro* analysis of cell behavior, or were carried out using specific datasets before the release of TCGA data. Therefore, the role of the Wnt pathway in GC remains insufficiently understood, and a revised overlook is thus necessary to assess whether updated data corroborate previous associations.

This study might contribute to advance our understanding of the role of non-canonical Wnt signaling in GC. First, the results confirm high expression of *WNT5A*, *FZD2*, *FZD5*, and *FZD7* in GC, while the analysis shows that *ROR2* and *FZD2* expression correlates with lower survival, and the combined *WNT5A*/*FZD2*/*ROR2* and *WNT5A*/*FZD7*/*ROR2* signatures strongly correlates with a worse prognosis. In contrast, despite the high levels of the canonical co-receptors *LRP5*/*LRP6* and the ligand *WNT2*, the latter previously reported to be overexpressed in GC (Katoh, 2001; Zhang et al., 2018) and likely involving Wnt/β-catenin signaling (Cheng et al., 2005), these genes were not correlated with either OS or RFS, even when evaluated as a signature. Second, the correlation between the non-canonical “signature” and parameters such as immune cell infiltration and mesenchymal markers, and the lower correlation between *LRP5*/*LRP6* and these same parameters, also favor a role of the non-canonical Wnt pathway in GC progression.

The analysis focused on *FZD2* and *FZD7* since these two Frizzled receptors are expressed in some GC cell lines and a murine model of GC (Flanagan et al., 2019). It has been reported that *FZD2* and *FZD7* are the primary frizzled receptors in the gastric epithelium, although FZD2 might be unable to compensate for the loss of FZD7 in this tissue (Flanagan et al., 2017). Also, FZD7 might be crucial for Wnt-driven gastric adenoma formation (Flanagan et al., 2019), most likely through the Wnt/β-catenin pathway (Li et al., 2018). However, some of this evidence is supported by proliferation assays in gastric organoids or expression of Wnt/β-catenin target genes, without rescue or competition experiments. Furthermore, other reports have shown that FZD7 can activate non-canonical Wnt signaling triggered by Wnt5a in different cellular contexts (Nishita et al., 2010; Anastas et al., 2014; Dong et al., 2017; Guan and Zhou, 2017). Moreover, Wnt5a can signal through FZD2 in GC (Hanaki et al., 2012).

Therefore, FZD2 might be unable to compensate for signal transduction from endogenous canonical Wnt ligands. However, it might compensate for other functions performed by FZD7, like signal transduction from non-canonical Wnt ligands, such as Wnt5a, likely secreted by TAMs (Zhao et al., 2013) or CAFs (Wang et al., 2016). Future experiments will be needed to corroborate these scenarios, including the shared and specific roles of FZD2 and FZD7 in GC. Notwithstanding, these frizzled receptors might transduce both canonical and non-canonical Wnt signals in GC, and thus pharmacological inhibition of these receptors acquires greater relevance (Koushyar et al., 2020). Also, a hexapeptide inhibitor of Wnt5a signaling, Box5 (Jenei et al., 2009), has been employed in some cancer studies (Tu et al., 2019; Luo et al., 2020), opening a new opportunity to explore the role of non-canonical Wnt signaling in GC.

It is important to note some limitations of this study. First, analysis at the protein level is limited to the data available in the TGCA, and other proteins might also be correlated with the non-canonical signatures, leading to different or additional conclusions. However, the data presented here suggest that KIT, MYH11, RICTOR, and claudin 7 should be studied in more detail. KIT, a type III receptor tyrosine kinase that binds the KIT ligand (KITLG; also known as stem cell factor ligand, SCF), activates many downstream pathways (Lennartsson and Rönnstrand, 2012), and thus it might converge with Wnt5a-mediated signaling to modulate several intracellular processes. Previous research has shown that KIT is altered in gastrointestinal stromal tumors (Hirota et al., 1998; Rubin et al., 2001). MYH11 encodes the smooth-muscle myosin heavy chain (also known as SMMHC). Mutations in MYH11 have been reported in gastric and colorectal cancers (Jo et al., 2018). It has been proposed that MYH11 might be related to stem cell differentiation defects, as well as disturbed energy balance (Alhopuro et al., 2008). In addition, KIT and MYH11 show a moderate correlation with immune cell infiltration, particularly CD4+ T cells and macrophages (Supplementary Figure 7C). Moreover, these proteins might activate different signaling pathways leading to cancer progression, as seen for KIT in hepatocellular carcinoma (Rojas et al., 2016). RICTOR (a component of the mTORC2 complex) expression is altered in several cancer types, and its involvement in cancer progression is likely explained by its well-known role in AKT signaling [reviewed in Gkountakos et al. (2018)]. However, mTOR-Wnt/β-catenin signaling crosstalk *via* Disheveled might also take place (Vadlakonda et al., 2013). It must be noted that Disheveled is involved in Wnt5a-mediated integrin adhesion turnover (Matsumoto et al., 2010); therefore, putative crosstalk mechanisms affecting Disheveled might also modulate Wnt5a signaling. Finally, claudin 7, a tight junction transmembrane protein, has been observed to be overexpressed in GC, correlating with a shorter OS (Jun et al., 2014). Interestingly, overexpression of claudin 7 leads to increased invasiveness in the gastric cell line AGS (Zavala-Zendejas et al., 2011). However, claudin 7 expression is either up- or downregulated in several cancer types, and dysregulated protein levels might interfere with its epithelial barrier function, leading to impaired tissue homeostasis (Wang et al., 2018). Moreover, cytoplasmic staining for claudin 7 has been reported in GC tissues (Johnson et al., 2005). Finally, the precise role of claudin 7 in GC might depend on the histological subtype (Johnson et al., 2005; Jun et al., 2014).

Second, the data presented here must be corroborated biochemically, both *in vitro* and *in vivo*. Third, this study needs to be extended at the histological level to fully corroborate the findings presented here. For instance, it is necessary to determine whether patients with high levels of *WNT5A*, *ROR2*, and *FZD2*/*FZD7* show increased levels of mesenchymal markers at the protein level. The same applies to proteins involved in the GO categories identified here as enriched in the non-canonical signature. Fourth, the result that a non-canonical Wnt signature is correlated with mesenchymal marker expression suggests that this pathway might be even more relevant than previously proposed. Future studies should address this correlation experimentally and compare the correlations observed for the non-canonical Wnt signatures to genes associated with pathways previously shown to be altered in GC (Shi, 2014). In this regard, the non-canonical Wnt signature correlations are similar to those seen for *SMO* (smoothened, frizzled class receptor), *EGF* (epidermal growth factor), and *ERBB4* (erb-b2 receptor tyrosine kinase 4) (Supplementary Figure 9). Finally, future studies should aim to understand the role of cytoplasmic Wnt components and effectors, such as Disheveled proteins, disheveled associated activator of morphogenesis 1 (Daam1), JNK, and others. Among these, Daam1 might have an important role in GC since high *DAAM1* expression correlates with lower survival, using terciles (Supplementary Figure 10). Of note, Daam1 has been associated with cancer progression in other tissues (Zhu et al., 2012; Wu et al., 2019; Rodriguez-Hernandez et al., 2020).

In summary, the results presented in this study corroborate previous insights about the role of Wnt5a and the non-canonical Wnt pathway in GC, helping to clarify the relevance of this Wnt signaling branch in GC from a prognostic perspective and extending previous knowledge, by showing that a signature associated with this pathway correlates with immune cell signatures and mesenchymal marker expression. Therefore, this study strengthens the need to better understand the biological and molecular processes modulated by this pathway in the homeostasis of the gastric tissue and cancer.

## Data Availability Statement

The datasets analyzed for this study can be accessed through the following websites: (a) cBioPortal, https://www.cbioportal.org (STAD Firehose Legacy dataset); (b) GEPIA (version 2.0), http://gepia2.cancer-pku.cn; (c) TIMER (version 1.0), https://cistrome.shinyapps.io/timer/; and (d) the Cancer Proteome Atlas, https://tcpaportal.org/tcpa/. Gene and protein lists are available in the Supplementary Information.

## Author Contributions

PA conceived and designed the study, and wrote, revised, and approved the submitted manuscript.

## Conflict of Interest

The author declares that the research was conducted in the absence of any commercial or financial relationships that could be construed as a potential conflict of interest.

## Footnotes

1 bioinformatics.psb.ugent.be/webtools/Venn/
