# Supplementary material for: A Non-canonical Wnt Signature Correlates With Lower Survival in Gastric Cancer
Source: Front Cell Dev Biol. 2021 Apr 1;9:633675. doi: 10.3389/fcell.2021.633675 (PMC8047116; doi:10.3389/fcell.2021.633675)
Supplement: Supplementary file 1 [file Image_1.PDF]

Supplementary Material

1 Supplementary Figures

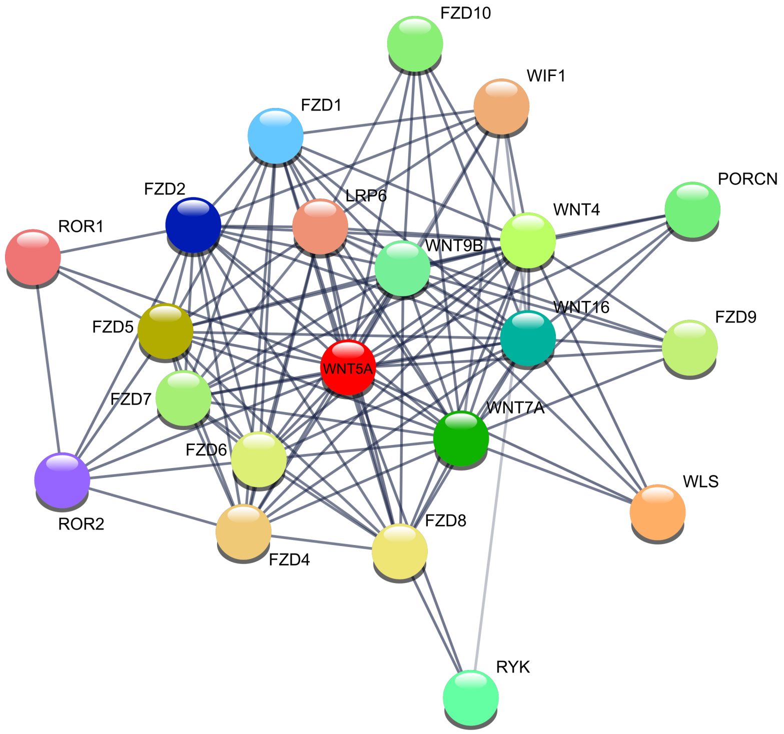

Supplementary Figure 1. Functional interactions for Wnt5a, according to the STRING database. The 20 top interactors are shown (see 'Materials and methods' for details).

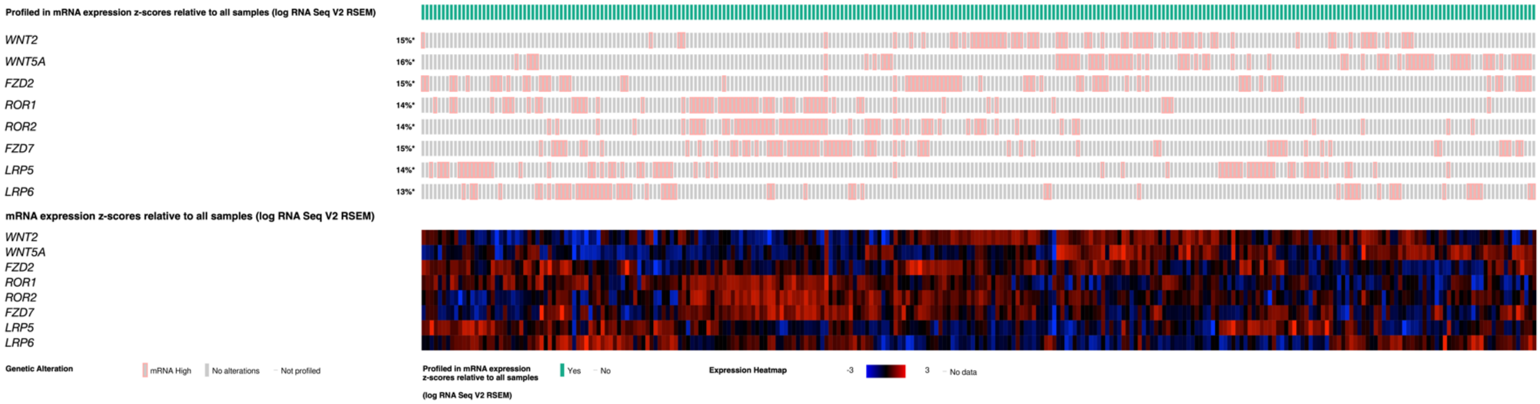

Supplementary Figure 2. Overlap between cases with high levels of the indicated genes (see 'Materials and methods' for details), and clustered using cBioPortal. The heatmap shown above suggests the existence of certain groups. For instance, one group indicates high levels of *FZD2*, *ROR1*, *LRP5,* and *LRP6*, while a second group shows high levels of *ROR1*, *ROR2,* and *FZD7*. Importantly, some cases with high *ROR2* expression also have either high *FZD2* or high *FZD7* expression. Cases with high levels of *WNT2* show greater overlap with cases with high *WNT5A* levels. For simplicity, only the samples with altered expression (as defined by the settings detailed in 'Materials and methods') are shown.

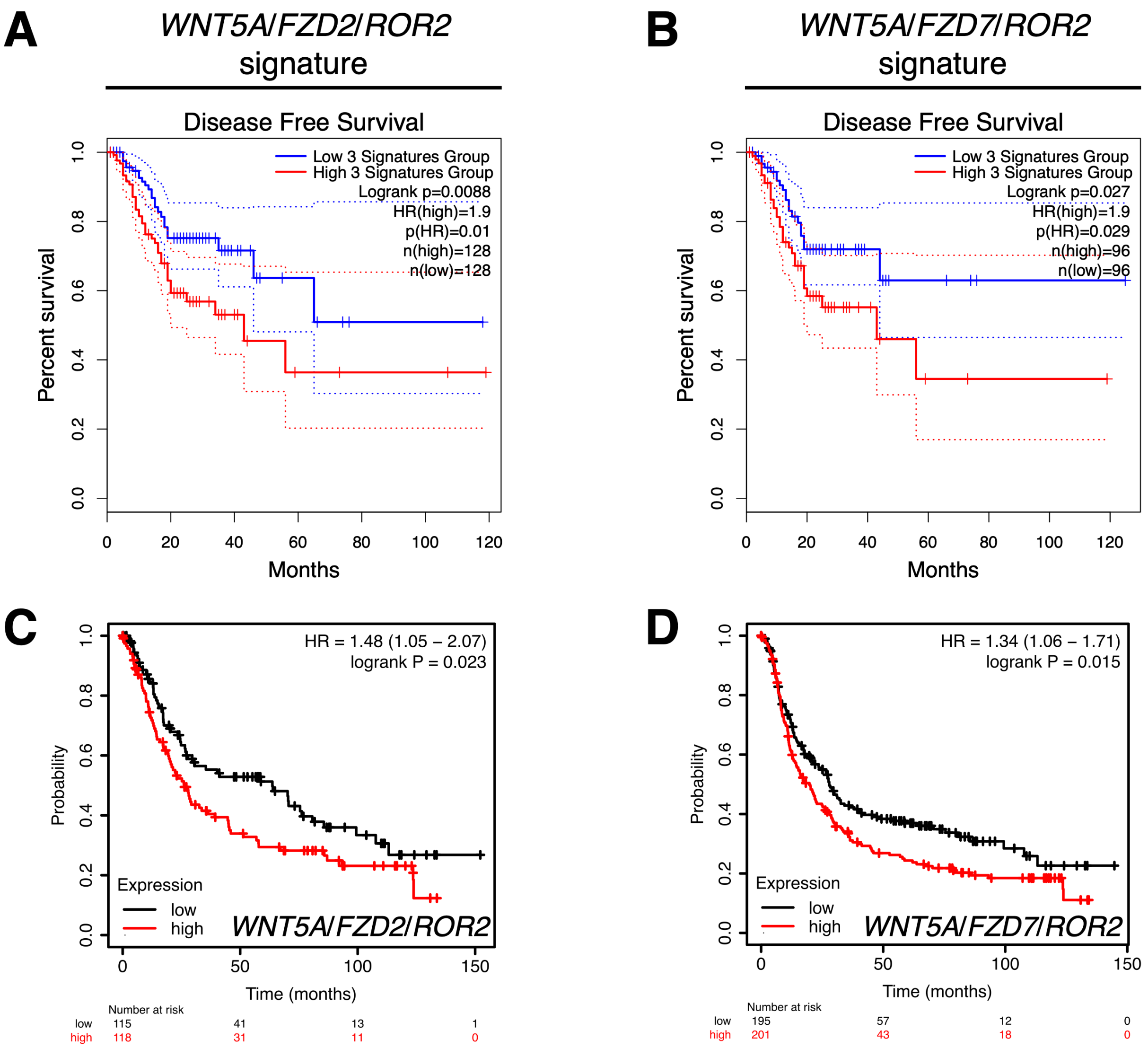

Supplementary Figure 3. (A, B) Survival plots (disease free survival; RFS) for the non-canonical Wnt signatures *WNT5A/FZD2/ROR2* (A; terciles) and *WNT5A/FZD7/ROR2* (B; quartiles). **(C, D)** Survival plots for the non-canonical Wnt signatures *WNT5A/FZD2/ROR2* (C) and *WNT5A/FZD7/ROR2* (D), obtained with KM Plotter, using terciles (see 'Materials and methods' for details).

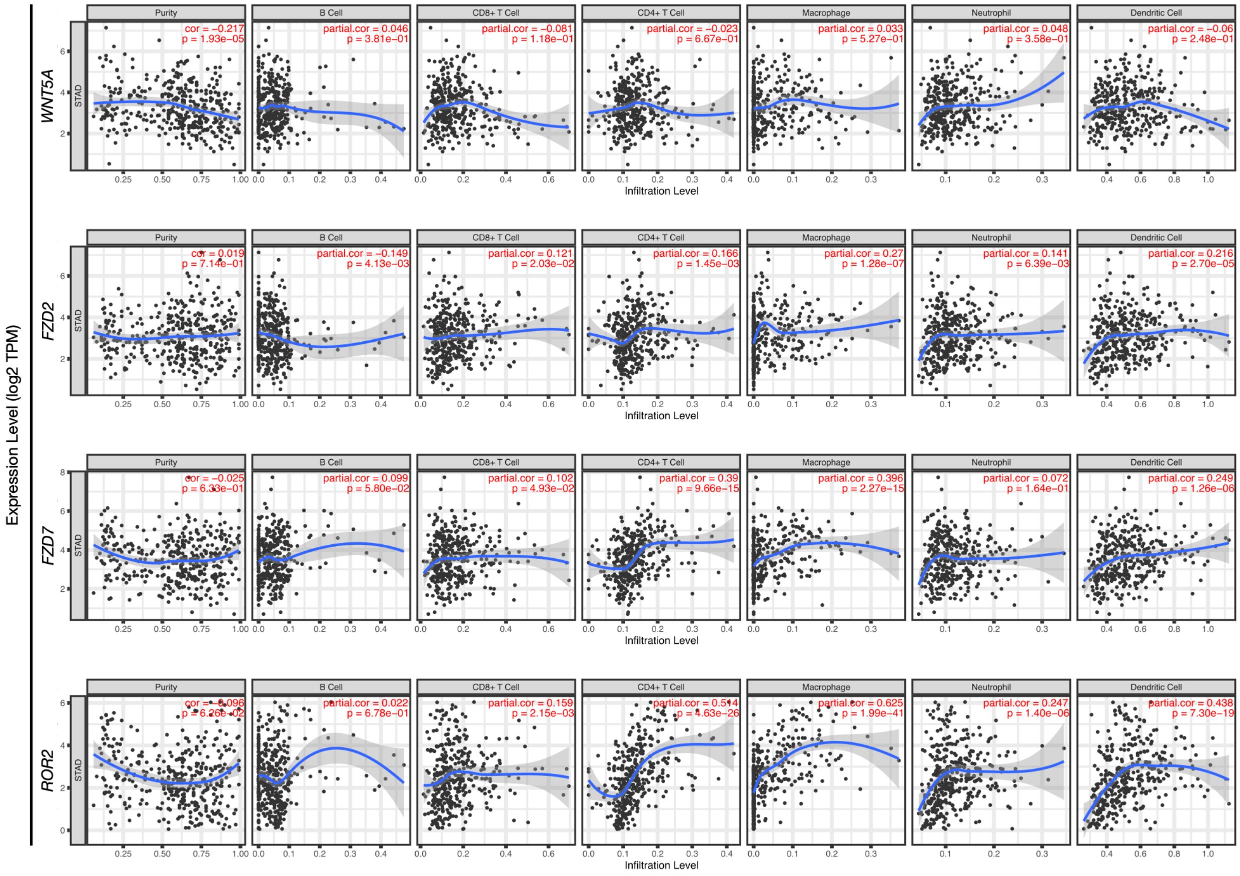

Supplementary Figure 4. Correlation between the *WNT5A*, *FZD2*, *FZD7*, and *ROR2* genes and immune cell infiltration. The plots were obtained using TIMER (version 1.0). Data was adjusted by tumor purity. The last row is also shown in Figure 3B.

Of note, the decreased correlation between the non-canonical signatures and immune cell signatures observed when using STAD tumor data (relative to normal data; Supplementary Table 3) remains to be clarified. A possible explanation is that immune cell infiltration proceeds through several mechanisms, with a small contribution of the non-canonical Wnt pathway. However, it must be noted that the correlations between these immune cell signatures and survival (Supplementary Table 4) were either weak or moderate (M2 signature), suggesting that other processes, such as EMT (see next figure), might contribute to the correlation between the non-canonical Wnt signatures and poor prognosis.

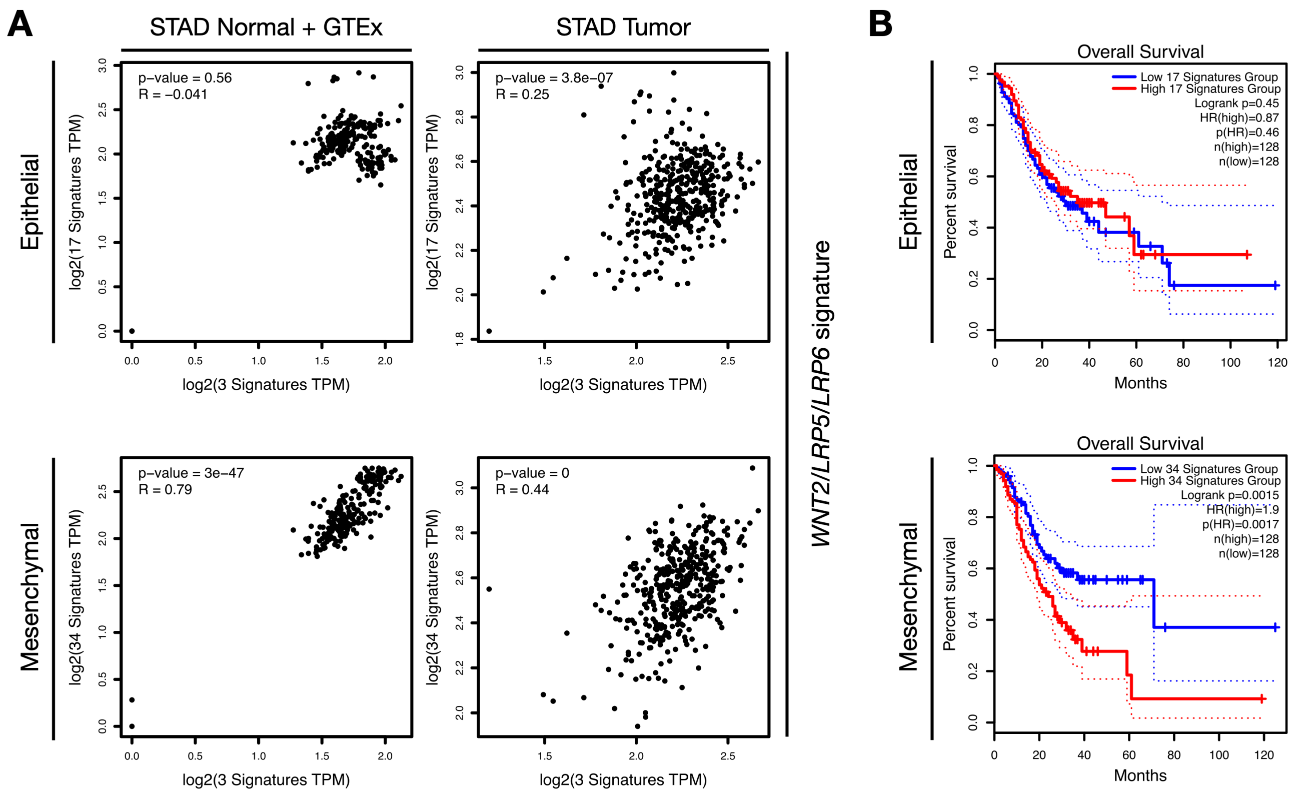

Supplementary Figure 5. (A) Plots showing the correlation between the Wnt/β-catenin (canonical) signature and the expression of epithelial (top) or mesenchymal (bottom) markers (listed in Supplementary Table S1). Normal (STAD Normal plus GTEx) or tumor (STAD Tumor) data was used for the analysis in GEPIA. **(B)** Survival plots, showing the effect of low (blue lines) or high (red lines) expression of the epithelial (top) or mesenchymal (bottom) markers on OS. Dotted lines show the 95% CI. The analysis was performed in GEPIA. Of note, the non-canonical Wnt signatures showed a noticeable correlation with the mesenchymal signature, even when using STAD tumor data, thus suggesting a more prominent role for EMT in the context of Wnt signaling.

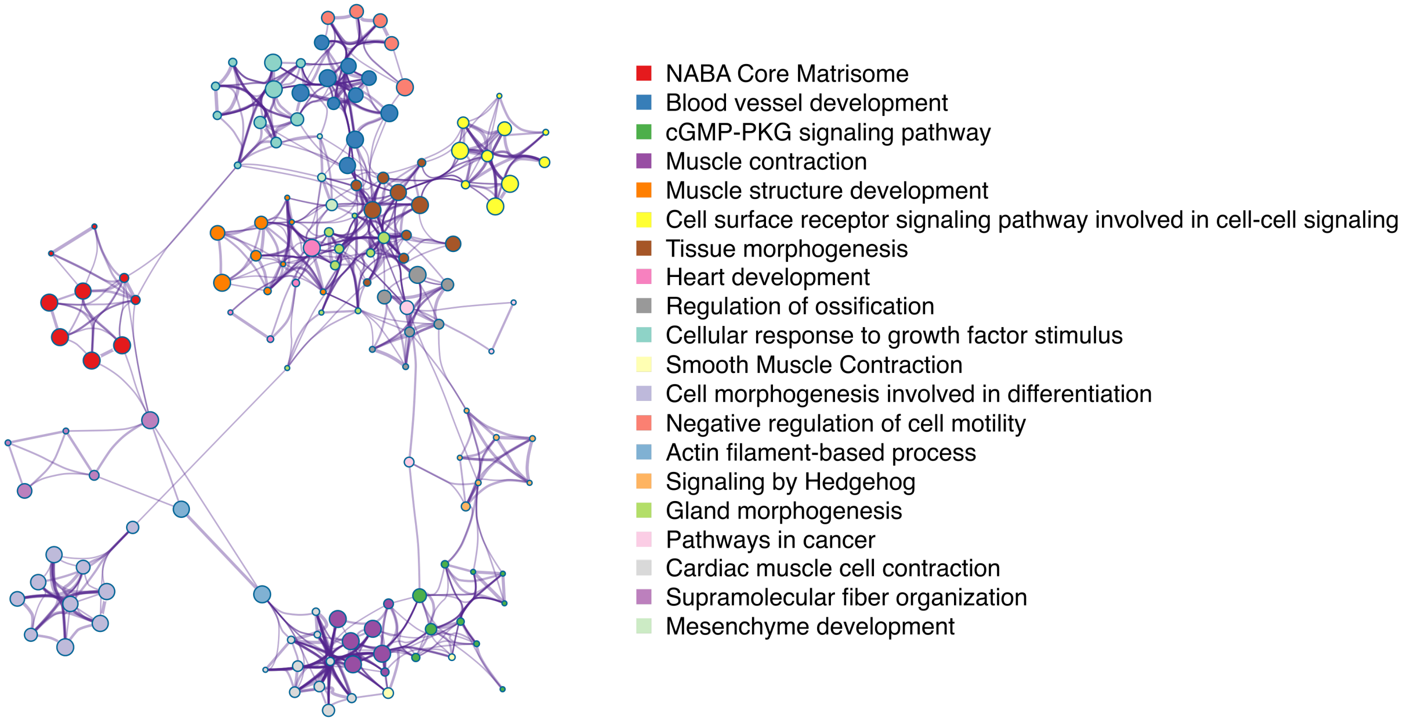

Supplementary Figure 6. Over-represented GO categories, according to Metascape, for the *ROR2*-correlated gene list. See the main text for details.

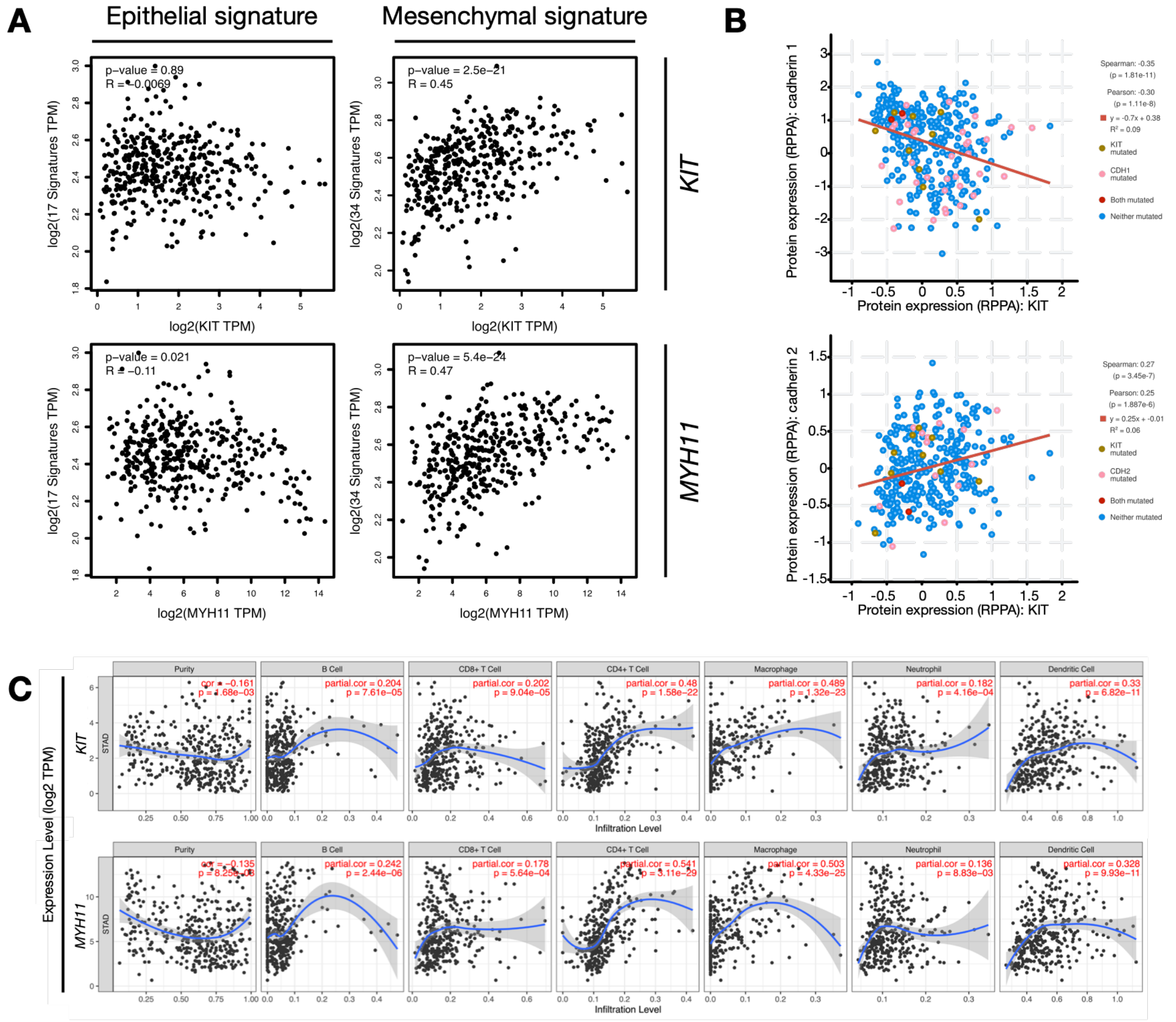

Supplementary Figure 7. (A) Correlation between *KIT* and *MYH11* expression and the epithelial and mesenchymal signatures. **(B)** Correlation between KIT protein (c-Kit) levels and cadherin 1 (E-cadherin; top) and cadherin 2 (N-cadherin; bottom) levels, using data from cBioPortal. **(C, D)** Correlation between *KIT* or *MYH11* gene expression and immune infiltration levels.

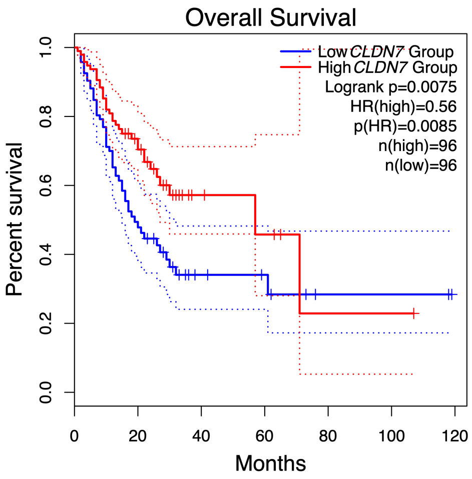

Supplementary Figure 8. Correlation between *CLDN7* expression and overall survival, using quartiles (n=96 per group).

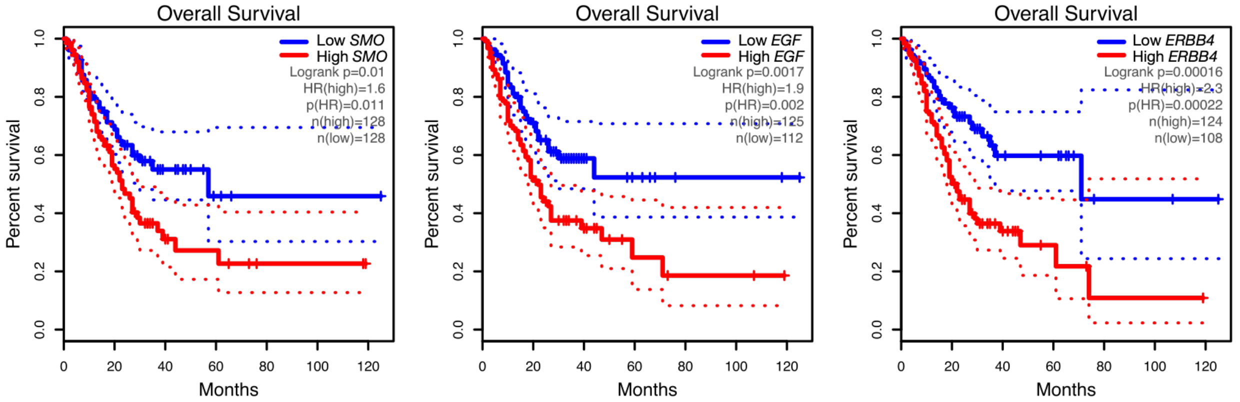

Supplementary Figure 9. Correlation between *SMO*, *EGF*, and *ERBB4* expression and overall survival, using terciles.

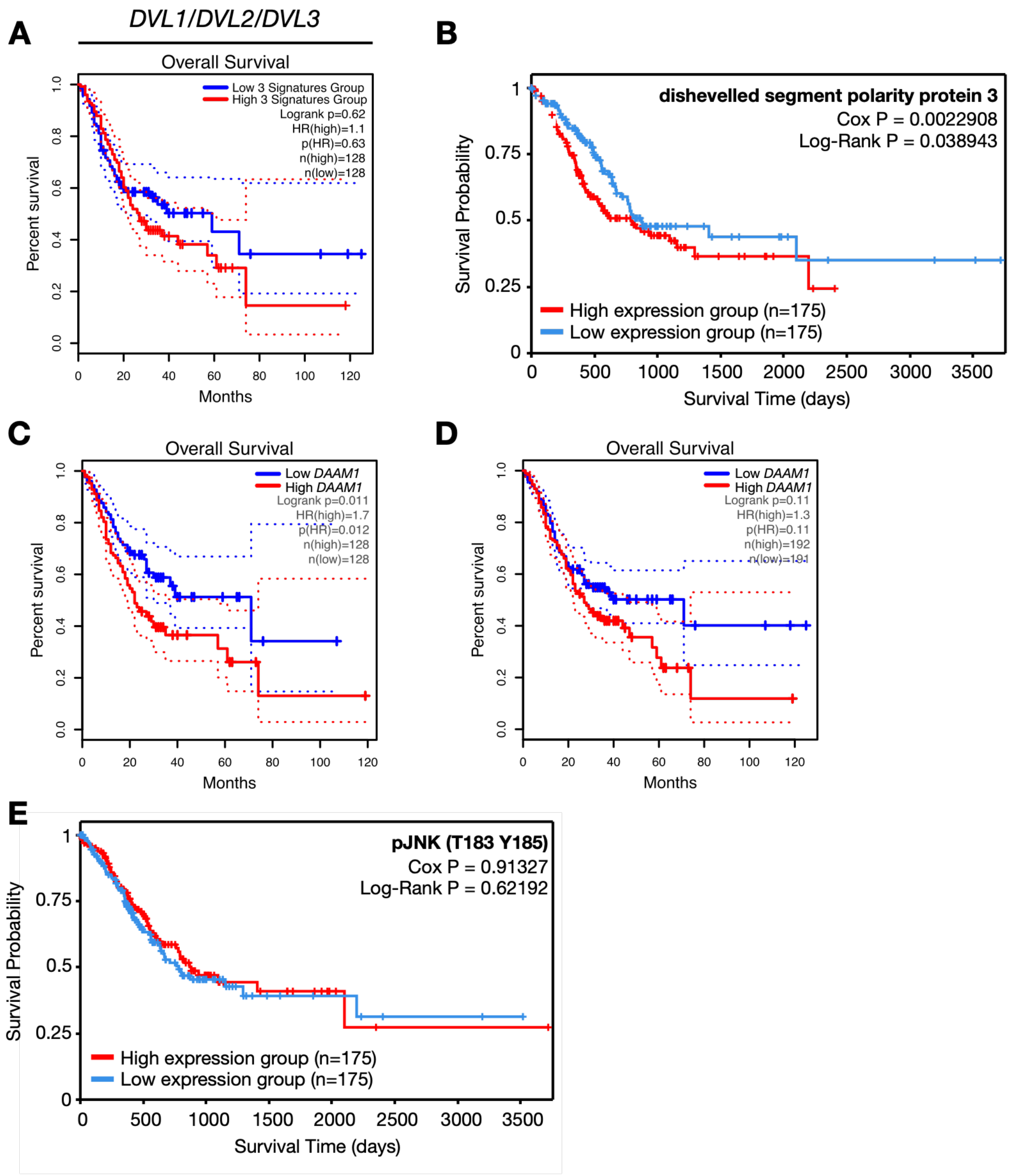

Supplementary Figure 10. (A) Correlation between *DVL1*, *DVL2* and *DVL3* expression and overall survival, using terciles. **(B)** Correlation between dishevelled segment polarity protein 3 (DVL3) protein expression and survival, using data from TCPA. **(C, D)** Correlation between *DAAM1* expression and overall survival, using terciles (C) or the median (D). **(E)** Correlation between JNK phosphorylation and survival, using data from TCPA.
